# Clozapine point of care testing in acute psychiatry: A precision approach to treatment resistant psychosis

**DOI:** 10.1192/j.eurpsy.2021.1216

**Published:** 2021-08-13

**Authors:** X. Boland, L. Dratcu

**Affiliations:** Inpatient Psychiatry, South London and Maudsley NHS Foundation Trust, London, United Kingdom

**Keywords:** clozapine, Point of care antipsychotic monitoring, Inpatient psychiatry, schizophrénia

## Abstract

**Introduction:**

Clozapine, the antipsychotic of choice for treatment-resistant schizophrenia, has a narrow therapeutic range and high interpatient variability in the dose-response relationship. Serum clozapine levels are essential both for therapeutic dosing and to monitor adherence. Use of venepuncture and prolonged result turnaround times with standard laboratory based methods for drug monitoring together contribute to the suboptimal use of clozapine.

**Objectives:**

A novel portable point-of-care (POC) device has been developed to measure whole blood clozapine concentrations using an automated homogenous immunoassay. It is as accurate and reliable as standard laboratory methods but only requires a drop of blood obtained by finger prick and can produce a result in minutes. We pioneered clozapine POC testing in the acute inpatient setting during the outbreak of the COVID-19 pandemic.

**Methods:**

We report on the use of POC clozapine testing in the management of 4 acutely psychotic patients with treatment resistant schizophrenia.

**Results:**

POC testing offered a more practical, less invasive and quicker alternative to conventional methods for monitoring of clozapine levels. Near immediate availability of clozapine levels expedited clinical decisions and helped ensure safe clozapine prescribing to severely unwell patients in a time of crisis. By facilitating patients’ early safe discharge from hospital, clozapine point of care testing also reduced length of hospitalisation.

**Conclusions:**

Point of care monitoring of other psychotropic medications in addition to clozapine brings about the prospect of personalised precision medicine for patients with severe mental illness, both in the acute setting and in the community.

